# The Role of Gut Microbiome Supplementation in COVID-19 Management

**DOI:** 10.7759/cureus.46960

**Published:** 2023-10-13

**Authors:** Mc Anto Antony, Siddharth Patel, Vipin Verma, Ravi Kant

**Affiliations:** 1 Department of Endocrinology, Diabetes and Metabolism, Medical University of South Carolina, Anderson, USA; 2 Department of Internal Medicine, Decatur Morgan Hospital, Decatur, USA; 3 Department of Internal Medicine, Medical University of South Carolina, Anderson, USA

**Keywords:** covid treatment, probiotic supplementation, covid-19, synbiotics, postbiotics, prebiotics, probiotics, gut microbiome

## Abstract

COVID-19, which is caused by the RNA virus, SARS-CoV-2, mainly affects the respiratory system and has a varied clinical presentation. However, several studies have shown that COVID-19 can also affect the gastrointestinal (GI) system. Patients can experience various GI symptoms, such as vomiting and diarrhea, and the virus has been detected in the stool samples of patients hospitalized with COVID-19. There have also been rare reports of COVID-19 presenting with isolated GI symptoms and lack of respiratory symptoms, and the virus has also been detected for prolonged periods in the fecal samples of COVID-19 patients. Major alterations in the gut microbiome in the form of depletion of beneficial organisms and an abundance of pathogenic organisms have been reported in the fecal samples of hospitalized COVID-19 patients. Although the US FDA has approved several drugs to manage COVID-19, their efficacy remains modest. So, there is a constant ongoing effort to investigate novel treatment options for COVID-19. Health supplements like probiotics, prebiotics, postbiotics, and synbiotics have been popularly known for their various health benefits. In this review, we have summarized the current literature, which shows the potential benefit of these health supplements to mitigate and/or prevent the clinical presentation of COVID-19.

## Introduction and background

**Figure 1:**
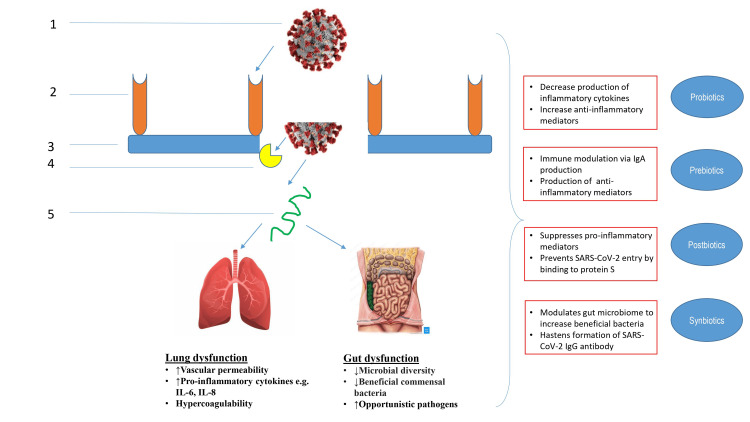
Schematic diagram illustrating the effects of SARS-CoV-2 on human lungs and gut. 1. SARS-CoV-2 virus; 2. ACE2 receptor; 3. Epithelium; 4. Transmembrane protease, serine 2 (TMPRSS2) receptor; 5. Viral RNA release. Source: Created by the authors.

COVID-19 can affect multiple organs, with the gastrointestinal (GI) system being one of the predominantly involved extra-pulmonary organ systems [1]. Clinical studies conducted even before the declaration of the COVID-19 outbreak as a global pandemic revealed the presence of GI symptoms such as diarrhea, nausea, and vomiting in COVID-19 patients [2,3]. Sometimes, patients present with isolated GI symptoms without respiratory involvement [4,5]. The virus has also been detected for prolonged periods in the fecal samples of COVID-19 patients [6,7]. Major alterations in the gut microbiome in the form of depletion of beneficial organisms and an abundance of pathogenic organisms have been reported in the fecal samples of hospitalized COVID-19 patients. At the phylum level, there was an alteration in two major phyla, Firmicutes and Bacteroidetes. At the family level, there was an abundance in pathogenic organisms such as Enterococcaceae, Coriobacteriaceae, Lactobacillaceae, Veillonellaceae, Porphyromonadaceae, and Staphylococcaceae and a decline in dominant families like Bacteroidaceae, Lachnospiraceae, and Ruminococcaceae, as well as Prevotellaceae and Clostridiaceae. At the genus level, COVID-19 patients showed an abundance of opportunistic pathogens like Enterococcus, Staphylococcus, Serratia, and Collinsella, as well as Lactobacillus, Parabacteroides, Lactococcus, Phascolarctobacterium, Odoribacter, Actinomyces, Methanobrevibacter and Akkermansia. At a species level, Enterococcus, which is one of the dominant genera in the gut microbiome of COVID-19 patients, was mainly comprised of *E. faecium* (8.4%), followed by *E. hirae* (5.5%), *E. faecalis* (1.8%) and *E. villorum* [8]. The above mounting evidence paved the way for researchers to study the relationship between the human gut and the SARS-CoV-2 antigen in-depth and elucidate ways to mitigate and/or prevent the dire clinical consequences of COVID-19 infection. Although several medicines (e.g., baricitinib, tocilizumab, and remdesivir) have been approved by the FDA for the management of COVID-19, their efficacy remains modest [9]. Several other drugs have failed to show consistent benefit in COVID-19 when studied in large-scale randomized controlled trials [9]. Hence, there is a huge unmet need for safe and efficacious treatment for COVID-19. Treatments to increase the number of beneficial gut bacteria to combat the gut microbiome changes caused by COVID-19 infection can potentially reduce and/or prevent the clinical consequences of COVID-19. Health supplements like probiotics, prebiotics, postbiotics, and synbiotics have been popularly known for their various health benefits. They are known to exert their actions via immunomodulation, which includes the production of antimicrobial peptides by intestinal cells [10], differentiation of T-helper cells [11], secretion of IgA, promotion of the growth of anti-inflammatory cytokines [12], and activation of splenocytes and dendritic cells [13]. They also play a beneficial role in metabolic diseases by improving insulin sensitivity in overweight and obese adults, lowering the risk of type 2 diabetes mellitus (T2DM) and obesity [14-16]. There is mounting evidence to suggest their beneficial role against COVID-19 infection. We searched PubMed for articles using the key terms: COVID-19, gut microbiome, probiotics, prebiotics, postbiotics, and synbiotics. The search dates ranged from 2002 through 2023, and the search included clinical trials, meta-analyses, randomized controlled trials, review articles, and case reports. The NIH reviewed treatment guidelines for COVID-19, and the expert consensus document from the International Scientific Association of Probiotics and Prebiotics (ISAPP) was reviewed to obtain the definitions of probiotic, prebiotic, postbiotic, and synbiotic. In this review, we have summarized all the available literature that points towards the beneficial role of the above supplements in COVID-19 disease. This has been illustrated in Table 1 and Figure 1.

**Table 1:** Mechanism and effects of supplements on human health.

Intervention	Definition	Examples	Effects
Probiotics	Live microorganisms that confer a benefit to the host [17]	Lactobacillus and Bifidobacterium	Lactobacillus releases angiotensin-converting enzyme (ACE)-inhibitory peptides from fermented milk [18, 19].
			Probiotics decrease pro-inflammatory cytokines [20] and enhance recruitment of anti-inflammatory mediators [21].
			Lactobacillus has favorable effect on metabolic syndrome, which is a strong risk factor for severe COVID-19 infection [22-24].
			Certain probiotics, psychobiotics, can alleviate neurological symptoms of COVID-19 [25-27].
Prebiotics	Substrates selectively utilized by host microorganisms conferring a health benefit to the host [28]	Fructans, oligosaccharides, arabinooligosaccharides, xylooligosaccharides, resistant starch, lactosucrose, lactobionic acid, galactomannan, psyllium, polyphenols [28-29]	Immune modulation via stimulation of secretion of IgA, prevention of overgrowth of bacteria, and promoting anti-inflammatory cytokines [12].
			Improvement of insulin sensitivity and obesity [14-16].
			Promote the growth of probiotics in the gut [30-32].
Postbiotics	Inanimate microorganism and/or their components that confer a benefit to the host [33]	Heat-killed Lactobacillus, peptides secreted by bacteria such as lactocepins and serpins	Immune modulation by suppressing pro-inflammatory mediators and enhancing anti-inflammatory mediators in the lung, liver, and plasma [34].
			Interferes with protein S binding to host cell [35, 36-38].
			Mitigates post COVID-19 mood and sleep issues via reduction of pro-inflammatory signals [27].
Synbiotics	Preparations consisting of live microorganisms and substrates selectively used by host microorganisms and confer a benefit to the host [39]	Combination of Bifidobacterium strains and galactooligosaccharides	Modulation of gut microbiome environment to increase beneficial microbes, Bifidobacterium and Lactobacillus, leading to lower incidence of enteritis and ventilator-associated pneumonia [40].
			Hastenes formation of SARS-CoV-2 IgG antibody [41].

## Review

Probiotic use in COVID-19 

Probiotics are comprised of "live microorganisms which when administered in adequate amounts confer a health benefit on the host" [17]. Probiotics are involved in host immune function modulation via various mechanisms, such as stimulating the production of antimicrobial peptides by intestinal Paneth cells [10] and differentiation of direct T-helper 17 cells in the small intestine [11]. The beneficial effect of a probiotic, Enterococcus faecium NCIMB 10415, has been seen against the enteropathogenic coronavirus transmissible gastroenteritis virus (TGEV), which causes severe gastroenteritis in newborn piglets [42]. The use of probiotics (especially Lactobacillus and Bifidobacterium) in children suffering from common acute upper respiratory tract infections (URTI) was associated with a significant reduction in the duration of illness episodes per person as well as a significant reduction in absenteeism from school/daycare, as demonstrated in a meta-analysis [43]. Treatment of mechanically ventilated patients with probiotics reduced the incidence of ventilator-associated pneumonia (VAP) [44, 45]. Results from human studies indicate that *L. rhamnosus* GG, *L. casei*, *L. plantarum*, *L. casei* strain Shirota, *B. lactis* Bb-12, and *B. longum* significantly reduced the prevalence of flu-like symptoms, upper respiratory infections, and antibiotic-associated diarrhea by up to 70% [46-48]. The probiotics *L. reuteri* ATCC 55730, *L. paracasei*, *L. casei* 431, *L. fermentum* PCC, and *B. infantis* 35624 promoted immunomodulatory changes during URTI and GI infection [49-51]. The above evidence clearly points towards the beneficial effect of probiotic use in respiratory and GI infections. Since the ACE2 receptor is known to be the gateway for entry of SARS-CoV-2, there is tremendous interest among researchers to elucidate a clinically meaningful role of modulating the receptor activity to serve as a potential therapeutic target against COVID-19 disease. Various studies have shown that Lactobacillus strains can release ACE-inhibitory peptides from fermented milk [18,19]. In human subjects with mild hypertension, administration of casein hydrolysate, a milk protein that contains tripeptides Val-Pro-Pro (VPP) and Ile-Pro-Pro (IPP), led to improvement in the vascular endothelial dysfunction via inhibition of ACE [18]. In another study, when probiotic strains (*Lactobacillus helveticus* KLDS.31 and *Lactobacillus casei* KLDS.105) were used to ferment bovine milk, it yielded milk-containing peptides with ACE-inhibitory activity [19]. When camel sausages were fermented with the novel probiotic *Lactococcus lactis *KX881782, there was a higher degree of antihypertensive activity via ACE inhibition [52]. Future studies are awaited to investigate the effect of probiotics on ACE receptors. COVID-19 is known to be associated with severe thrombosis and coagulopathy [53], and therefore, modulating the immune-coagulative response in the host may serve as a potential therapeutic target against COVID-19. Intranasal administration of the probiotic lactic acid bacteria (LAB) in mice with pneumococcal infection led to control of inflammation and hemostatic changes [54]. As mentioned above, cytokine storm is an exaggerated inflammatory response leading to the dreadful acute respiratory distress syndrome (ARDS) complication in COVID-19; therefore, preventing the excess cytokine release can serve as a potential therapeutic target [55]. Pre-clinical data suggests that the use of probiotics against the influenza virus offers beneficial effects via immune modulation through increased alveolar macrophage cells and decline in pro-inflammatory cytokines [20], enhanced recruitment of anti-inflammatory mediators like Treg cells and IL-10 [21], and improved lung transcriptional response against the virus [56].

It is well known that metabolic diseases, such as obesity and DM, as well as cardiovascular diseases, are strong risk factors for severe disease and higher mortality among patients with COVID-19 infection [57-59]. Studies have shown that probiotics have a favorable effect on glucose levels, blood pressure, low-density lipoprotein (LDL) cholesterol, and BMI [22]. *Lactobacillus coryniformis* CECT5711 demonstrated an improvement in endothelial dysfunction and vascular oxidative stress in obese mice that were exposed to a high-fat diet (HFD). It also reduced mice's basal glycemia and insulin resistance [23]. When hypertensive rats were administered the probiotic *Lactobacillus fermentum* CECT5716 or a combination of *L. coryniformis* CECT5711 and *L. gasseri* CECT5714 (LC9) in a 1:1 ratio, there was a reduction in systolic blood pressure, reversal of the impaired aortic endothelium-dependent relaxation to acetylcholine, and a reduction in cardiac and renal hypertrophy [24].

Clinical studies that have been conducted to date have indeed shown the benefit of using probiotics as an adjuvant measure in the management of COVID-19 disease [60-63]. In a randomized, blinded, controlled clinical trial conducted in Mexico, COVID-19 patients were randomized to a control group (CG) (n = 40) that received the standard hospital diet and hospital treatment and to an intervention group (IG) (n = 40) that in addition to the above, received the following intervention; 1) B-complex (10 mg of cyanocobalamin, 100 mg of thiamin, and 100 mg of pyridoxine) administered intramuscularly every 24 h for the first five days, 2) Nutritional support system (NSS) powder containing spirulina maxima, few trace elements and vitamins, and 3) probiotic Saccharomyces boulardii (SB) 500 mg daily for six days orally. When compared to the CG, patients in the IG had an increased survival rate (82.5% vs 97.5%) and a decreased mortality rate (17.5% vs 2.5%) that was statistically significant p = 0.027 [60]. In a single-center quadruple-blinded RCT, 300 outpatients with symptomatic COVID-19 were prescribed a 30-day course of probiotics *Lactiplantibacillus plantarum* and *Pediococcus acidilactici*. Remission was seen in 53% of the probiotic group compared to 28% in the placebo group [61].

In an RCT of 100 COVID-19 patients hospitalized with moderate pneumonia (confirmed by computed tomography and involving an area of no more than 75% of the lung), the use of Bifidobacterium bifidum 1 (5108 KOE) showed an improvement in the well-being and a reduction in the duration of the diarrheal syndrome [62].

Similarly, in another prospective RCT conducted in Rome, a mixture of three probiotics strains, *Bifidobacterium lactis* LA 304, *Lactobacillus salivarius* LA 302, and *Lactobacillus acidophilus* LA 201, were administered to COVID-19 patients with interstitial pneumonia, there was a decline in fecal calprotectin (35% in probiotic group vs. 16% in control group) and C-reactive protein (72.7% in probiotic group vs. 62% in control group) [63]. Interestingly, none of the above-mentioned probiotics led to any serious adverse events in the human and animal study populations.

Following COVID-19 disease, a significant number of patients experience cognitive issues, depression, anxiety, and memory loss [64]. Research suggests using certain probiotics, also known as psychobiotics, in sufficient amounts can alleviate neurological symptoms [25-27]. Evidence shows that *Lacticaseibacillus rhamnosus* and *Lacticaseibacillus casei* secretes the neurotransmitter GABA, which exerts an anti-depressive effect via its action on the GABA A and B receptors. Similarly, GABA secreted by *Levilactobacillus brevis* induces sleep. Secretion of serotonin and norepinephrine (NE) by *Lactobacillus hevleticus* can help improve cognitive impairments like memory and loss of concentration via modulation of the brain serotonin and NE system, and HPA axis. *Limosilactobacillus reuteri* secretes histamines that lower the expression of proinflammatory cytokines and thereby prevent a decline in hippocampal brain-derived neurotrophic factor (BDNF), a biomarker of mental health [27].

Prebiotic use in COVID-19

Prebiotic is "a substrate that is selectively utilized by host microorganisms conferring a health benefit" [28]. It commonly includes fructans, oligosaccharides, arabinooligosaccharides, isomaltooligosaccharides, xylooligosaccharides, resistant starch, lactosucrose, lactobionic acid, galactomannan, psyllium, polyphenols, and polyunsaturated fatty acids. Prebiotics play a role in immune modulation by stimulating the secretion of IgA, preventing the overgrowth and translocation of bacteria, and promoting the growth of anti-inflammatory cytokines [28,29]. A novel polysaccharide WSRP-1b extracted and purified from Kushui rose waste demonstrated immunomodulatory activity by increasing the production of ROS, nitric oxide (NO), and cytokines such as IL-6 and enhancing the phagocytic action of macrophages [65]. The US FDA approved the use of Chinese yam polysaccharide poly (lactic-co-glycolic acid) to develop vaccine delivery systems due to its demonstrated immune regulation and immune potentiation properties [66]. Prebiotics have been shown to be beneficial in not only improving common GI symptoms like diarrhea and constipation [67] but also in improving insulin sensitivity [14] in overweight and obese adults, lowering the risk of type 2 diabetes mellitus (T2DM) [15, 68], obesity and hyperlipidemia [16, 69]. There is also evidence showing a lower risk of URTI with the use of galactans and fuctans in infant formula [67]. In a systematic review of 12 studies with 688 participants, authors found that patients who received supplementation with prebiotics and probiotics had higher influenza hemagglutination inhibition antibody titers after vaccination. Although there was high heterogeneity across the studies, the results suggest a method to improve the vaccine efficacy [70]. There were also no adverse events reported in the individual studies except for one study in which the adverse events were either insignificant or comparable to the control group. Lymphocytopenia was found to be one of the most common findings in COVID-19 patients at the time of hospital admission [71], and the elevated levels of the proinflammatory cytokines such as IL-6 and IL-8 in patients with severe disease also correlated with lymphocytopenia [72]. Prebiotics may be of some benefit in this regard via their positive effect on lymphocytes. This was shown in a mice study wherein there was a substantial increase in CD8+ counts in the cecum when the dietary sugar beet fiber was fed to mice [73]. Glycan, a prebiotic, is also known to play a role in immune regulation and is one of the key regulators of antibody effector functions [74]. It is known that both the SARS-CoV-2 antigen and its main target ACE2 are highly glycosylated [75] and a study analyzing site-specific N-linked glycosylation of SARS S glycoprotein showed site occupancy by up to 10 different glycans, known as glycoforms [76]. This finding suggests the potential role of glycan against SARS-CoV-2, although further studies are needed. Human milk is universally considered the first line of nutrition for newborn infants, and it contains all the essential nutrients required for the infant, including human milk oligosaccharides (HMO) that possess various important functions, such as altering the gut microbial composition by promoting the growth of *Bifidobacterium bifidum* [77]. HMO also possesses anti-viral activity by mimicking the structure of viral receptors, thereby blocking viruses' adherence to target cells [78]. At present, HMO activity against SARS-CoV-2 is not known. However, there is evidence showing that milk produced by COVID-19-positive women does not represent a significant source of SARS-CoV-2 transmission, which is likely due to the passive immunity achieved via a robust anti-receptor binding domain (RBD) IgA response [79-81]. Future studies are warranted to investigate the potential use of extracted milk IgA for therapeutic use against SARS-CoV-2. Strong clinical evidence shows that prebiotics can promote the growth of certain probiotics like Lactobacillus and Bifidobacterium in the gut [30-32]. Currently, limited clinical data shows the benefit of using prebiotics against COVID-19 infection. Nevertheless, as indicated above, substantial indirect evidence points towards the benefit of prebiotic use to target the various pathophysiological mechanisms involved in a patient with SARS-CoV-2 infection. The results are promising, and future studies are needed to thoroughly investigate the impact of prebiotic use on various clinical parameters in COVID-19 disease. 

Postbiotic use in COVID-19

Postbiotic is defined as a "preparation of inanimate microorganisms and/or their components that confers a health benefit on the host" [33].

Postbiotics have been shown to exert immune-modulating effects in the host, as seen in the ability of heat-killed *Lactobacillus rhamnosus* GG to suppress proinflammatory mediators and enhance anti-inflammatory mediators in multiple organs, such as lung, liver, and plasma [34]. Postbiotics also exert other immunoregulatory effects, such as activating splenocytes and dendritic cells and producing TNF-a, IL-6, and IL-10 [13, 82].

Two separate studies were performed on elderly volunteers residing in nursing homes to evaluate the effect of heat-killed Lactobacillus on immune function [83, 84]. Both study designs were similar in that participants were randomly assigned to receive either the postbiotic jelly (heat-killed Lactobacillus) or the placebo jelly, followed by administering the influenza vaccine a few weeks later. Although there was no significant difference in the immune parameters between the postbiotic-treated group and the placebo group, there was an improvement in the hemagglutination inhibition (HI) titers against influenza antigens [83] and improved antibody response to type A/H1N1 influenza vaccine [84]. Both of the above studies did not report any adverse events in the intervention group.

Protein S, present in the SARS-CoV-2 virus, plays a key role in facilitating the entry of the virus into the host cell [35]. Hence, interfering with this pathway can prevent host cell infectivity and subsequent disease. A study showed that the metabolic products of probiotics, namely Plantaricin BN, D, W, and JLA-9, exhibited activity against the RBP on protein S [85]. Another study showed that glycocin F from *Lactococcus lactis *and *lactococcin*e G from *L. plantarum* showed a high binding affinity towards the SARS-CoV-2 protein S [36]. In an in-silico experiment, four different probiotic-derived peptides, subtilisin (Bacillus amyloliquefaciens), curvacin A (*Lactobacillus curvatus*), sakacin P (*Lactobacillus sakei*), and *lactococcin* Gb (*Lactococcus lactis*), showed high affinity to bind with protein S or RBD of S1 of SARS-CoV-2 and human ACE2 receptor. The above findings indicate the additional advantage of using probiotics due to their peptide derivatives to control the SARS-CoV-2 infection [37].

There is also evidence showing the influence of postbiotics on mental health following COVID-19 disease [27]. Some probiotics can produce bioactive molecules or postbiotics that act via separate pathways to confer a mental health benefit. Lacticaseibacillus paracasei secretes lactocepins that lower the migration of inflammatory cytokines, which are released during the viral encounter [27]. Bifidobacterium longum secretes serpins that enter the brain via the neural route and reduce the proinflammatory signals [27]. Lactobacillus gasseri secretes gassericins that promote sleep and improve the gut microbial composition [27]. EPS secreted by Lactobacillus kefiranofaciens has immunomodulatory properties that may prevent the hyperactivity of the HPA axis [27]. These findings suggest the beneficial role of using postbiotics to help mitigate post-COVID-19 mood and sleep issues, although further studies are awaited to fully elucidate their role in this aspect.

Synbiotic use in COVID-19

A synbiotic is defined as "a mixture comprising live microorganisms and substrate(s) selectively utilized by host microorganisms that confers a health benefit on the host" [38]. In an RCT conducted in rural India, when an oral synbiotic mixture containing Lactobacillus plantarum and fructooligosaccharide was administered to newborn infants, there was a significant reduction in the primary outcome (combination of sepsis and death) in the intervention arm and a significant reduction in culture-positive and culture-negative sepsis as well as lower respiratory tract infection [39]. In another RCT, mechanically ventilated septic patients in the ICU were administered a synbiotic mixture of Bifidobacterium breve strain Yakult, Lactobacillus casei strain Shirota, and galactooligosaccharides within three days of admission. When compared to the placebo arm, the treatment arm showed a significantly lower incidence of enteritis and ventilator-associated pneumonia (VAP) and a significantly higher fecal content of Bifidobacterium and Lactobacillus [40]. These findings suggest that synbiotics can modulate the gut microbial environment to exert various beneficial effects on the host, and neither of the above studies reports any adverse events in the intervention/treatment arm. There is some evidence showing a beneficial effect of synbiotics in COVID-19 patients. A study conducted in hospitalized COVID-19 patients in Hong Kong showed that administration of SIM01 synbiotic (a novel formula of Bifidobacterium strains, galactooligosaccharides, xylooligosaccharide, and resistant dextrin) hastened the formation of the SARS-CoV-2 IgG antibody and led to a reduction in the nasopharyngeal viral load and restoration of gut dysbiosis [41]. Mild adverse events (dizziness, tinea infection, and hypertension) were reported in only three out of 25 patients in the treatment arm. In a randomized placebo-controlled trial, when hospitalized COVID-19 patients were administered a synbiotic capsule containing multi-strain probiotics like *Lactobacillus rhamnosus*, *L. helveticus*, *L. casei*, *Bifidobacterium lactis*, *L. acidophilus*, *B. breve*, *L. bulgaricus*, *B. longum*, *L. plantarum*, *B. bifidum*, *L. gasseri*, and *Streptococcus thermophilus* (10x9 CFU), and fructooligosaccharides (a prebiotic agent), there was a significant reduction in serum IL-6 levels and WBC count, suggesting a potential role of synbiotic use as an adjuvant therapy to modulate the inflammatory responses seen in COVID-19 infection [86]. Henceforth, it is worthy of recognizing the potential role of using synbiotics as an adjuvant measure in combating severe infectious diseases, including COVID-19.

## Conclusions

There is data to suggest the beneficial role of using health supplements like probiotics, prebiotics, postbiotics, and synbiotics against COVID-19 disease. Studies conducted in humans and animals have shown their ability to exert various immuno-modulating actions, such as increasing secretory IgA and ROS levels (prebiotics), inhibiting protein S action (postbiotics), promoting the production of SCFAs (high fiber diet), reducing the proinflammatory WBC and IL-6 levels (synbiotics), and blocking ACE2 receptor activity (probiotics). They have also shown a benefit in improving certain metabolic parameters, such as obesity and hyperglycemia. There is no recommendation for or against the use of the aforementioned health supplements in the management of COVID-19 disease. Nevertheless, substantial direct and indirect evidence shows their benefit by targeting the various pathophysiological mechanisms involved in a patient with SARS-CoV-2 infection. Worldwide, they have been and/or are being utilized as an adjuvant measure in the treatment protocol for COVID-19 patients. Future well-designed and larger trials are needed to continue enhancing our understanding and accumulating stronger evidence before these supplements can be considered standard of care in COVID-19 disease management.
